# Evaluation of surface and shallow depth dose reductions using a Superflab bolus during conventional and advanced external beam radiotherapy

**DOI:** 10.1002/acm2.12269

**Published:** 2018-02-10

**Authors:** Jihyung Yoon, Yibo Xie, Rui Zhang

**Affiliations:** ^1^ Physics and Astronomy Louisiana State University Baton Rouge LA USA; ^2^ Mary Bird Perkins Cancer Center Baton Rouge LA USA

**Keywords:** external beam radiotherapy, patient safety, shallow depth dose, shielding, Superflab bolus, surface dose

## Abstract

The purpose of this study was to evaluate a methodology to reduce scatter and leakage radiations to patients’ surface and shallow depths during conventional and advanced external beam radiotherapy. Superflab boluses of different thicknesses were placed on top of a stack of solid water phantoms, and the bolus effect on surface and shallow depth doses for both open and intensity‐modulated radiotherapy (IMRT) beams was evaluated using thermoluminescent dosimeters and ion chamber measurements. Contralateral breast dose reduction caused by the bolus was evaluated by delivering clinical postmastectomy radiotherapy (PMRT) plans to an anthropomorphic phantom. For the solid water phantom measurements, surface dose reduction caused by the Superflab bolus was achieved only in out‐of‐field area and on the incident side of the beam, and the dose reduction increased with bolus thickness. The dose reduction caused by the bolus was more significant at closer distances from the beam. Most of the dose reductions occurred in the first 2‐cm depth and stopped at 4‐cm depth. For clinical PMRT treatment plans, surface dose reductions using a 1‐cm Superflab bolus were up to 31% and 62% for volumetric‐modulated arc therapy and 4‐field IMRT, respectively, but there was no dose reduction for Tomotherapy. A Superflab bolus can be used to reduce surface and shallow depth doses during external beam radiotherapy when it is placed out of the beam and on the incident side of the beam. Although we only validated this dose reduction strategy for PMRT treatments, it is applicable to any external beam radiotherapy and can potentially reduce patients’ risk of developing radiation‐induced side effects.

## 1 INTRODUCTION

The number of cancer survivors has been increasing. It is estimated that cancer survivors will account for about 5.4% of the US population in 2024, and approximately half of all cancer patients receive radiotherapy as a part of their treatments (http://www.cancer.gov). There is a growing concern about patient safety because side effects induced by cancer treatments may remain and seriously affect their quality of life for many of these survivors. While the goal of radiotherapy is to deliver a highly conformal dose to the tumor area only, normal tissues outside the target also receive radiation doses including medium to high dose adjacent to the target, scatter and leakage doses from the treatment machine, and scatter dose within the patient. These normal tissue doses can cause a spectrum of acute and chronic radiogenic side effects for the patients.^1^, ^2^, ^3^, ^4^, ^5^, ^6^, ^7^, ^8^, ^9^, ^10^, ^11^, ^12^, ^13^, ^14^ Epidemiologic studies indicate the majority of second cancers occurred in the low‐ or intermediate‐dose areas,^15^, ^16^ and current data support linear‐no‐threshold dose‐risk model indicating a finite risk of developing second cancer even for the lowest radiation dose.^17^, ^18^ The advanced radiotherapy techniques like intensity‐modulated radiotherapy (IMRT) will increase the low‐dose volume because of beam modulations and can increase the risk of developing second cancers.^19^, ^20^

Over the years, people have been trying to reduce normal tissue doses during radiotherapy, and external shielding is one of the effective approaches to reduce the scatter and leakage doses from the treatment machine. Multiple studies^21^, ^22^, ^23^, ^24^ reported lead blocks can be used to limit fetal dose during radiotherapy for pregnant patients; lead sheets had also been shown to reduce scatter radiation to the contralateral breast or heart during breast cancer radiotherapy.^25^, ^26^, ^27^, ^28^, ^29^ Most of the previous studies chose a high‐density metal as the shielding material, and holders usually had to be used to support the heavy shielding blocks, which makes the procedure expensive, troublesome, and time consuming. Occasionally, the shielding block may be dropped by accident and could hurt the patient or staff. Although some specially designed shielding device made of a very thin lead sheet can overcome some of these problems, it has the limited availability, does not match the body surface, and additional material has to be used to fill the gap between the body and the shielding device to reduce the lateral scattering dose.^30^ One study reported that placing a Superflab bolus on the surface of the contralateral breast during external radiotherapy could reduce the surface dose under the bolus.^31^ This is an attractive alternative because the Superflab bolus is widely available, has a much lower cost compared with lead sheets, and can be put directly on the patient and conform to the body shape very well. The effect of the bolus was only evaluated for the conventional tangential breast radiotherapy in that study and only 1‐cm Superflab bolus was used.^31^

In this study, we investigated this bolus effect in more details: the bolus was placed in the field and out of the field, on the incident and exit sides of the radiation beam; various bolus thicknesses were used; both surface and shallow depth doses at different distances from beam axis were investigated; both static and IMRT beams were tested; and dose reductions for advanced radiotherapy techniques in clinically realistic situations were also evaluated.

## 2 MATERIALS AND METHODS

### 2.A Surface dose evaluation

**Figure 1:**
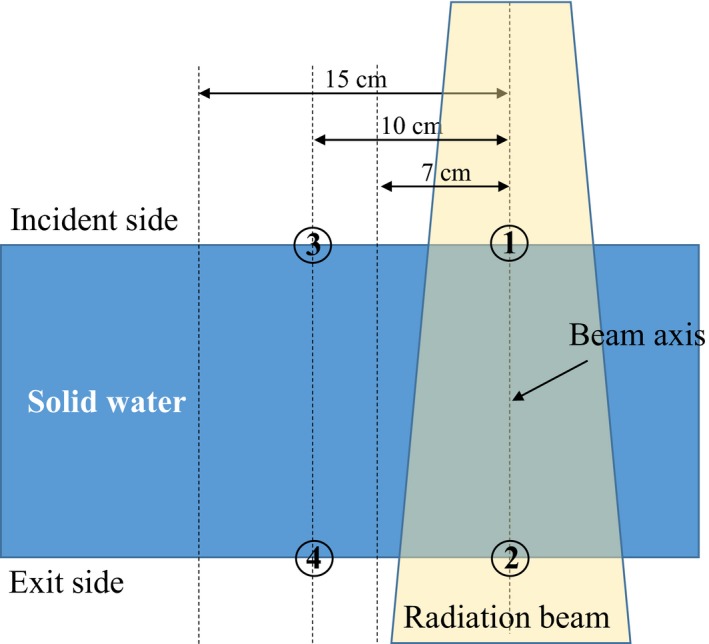
A solid water phantom used for bolus effect evaluation. Surface points 1 and 2 were on the beam axis and in the field, and points 3 and 4 were 10 cm away from the beam axis and out of the field. For the radiation beam shown in the figure, points 1 and 3 were on the incident side, and points 2 and 4 were on the exit side of the beam. We also measured doses 7, 10, and 15 cm away from the beam axis and at various depths.

A stack of solid water phantoms was used to evaluate the skin dose with and without a Superflab bolus (Radiation Products Design Inc., Albertville, MN, USA) placed in the field or out of the field (Fig. 1). The Superflab boluses used in this study are commercial products and made of synthetic gel which is water equivalent. They have the same size (30 × 30 cm^2^) but the thickness varies. The bolus can be cut to any shape to fit the patient's contour for any radiotherapy when necessary, although we did not cut them in this study. Thermoluminescent dosimeters (TLDs) were placed at the measurement points shown in Fig. 1. Measurement points 1 and 2 were on the beam axis and in the field, and points 3 and 4 were 10 cm away from the beam axis and out of the field. For in‐field measurements, 6 and 10 MV open beams with 10 × 10 cm^2^ field size at the isocenter and IMRT beams with a maximum multileaf collimator (MLC) opening less than 10 × 10 cm^2^ at the isocenter were used, and 100 monitor units (MUs) were delivered using Elekta Infinity linac (Elekta Corporation, Stockholm, Sweden). The source‐to‐surface (SSD) was 90 cm and the beam isocenter was located at 10 cm depth and 10 cm away from the edge of the phantom (Fig. 1), and only 1‐cm Superflab bolus was used in the in‐field measurements and was placed above point 1 or 2. For out‐of‐field measurements, the same open photon beam with 2000 MUs and IMRT beam with 1982 MUs were delivered to accumulate enough TLD readings. Boluses of 0.5, 1, 1.5, and 2 cm thicknesses were used in the out‐of‐field measurements because we are more interested in dose reductions, and the bolus was placed out of the beam with its edge aligned with the field edge.

Each TLD dosimeter contained approximately 45 mg TLD‐100 (LiF:Mg,Ti) powder sealed in a cellophane packet with a volume of approximately 1 × 1 × 0.2 cm^3^. After TLD measurements, we performed TLD calibrations by placing TLD packets in solid water phantoms, delivering a known dose to the TLD packet using the radiation beam of a specified energy, and recording the TLD readings. This was repeated for several dose levels and a calibration curve was created based on the readings. The TLD packets were read using a REXON UL‐320 Reader (Rexon Components, Inc., Beachwood, OH, USA). The TLD powder in each packet was divided into three samples of approximately 15 mg each, and the three samples were used to determine the mean dose and standard deviation (SD) of the mean for each TLD packet. The uncertainty of the dose measured by each TLD is ≤4% according to the literature.^32^, ^33^

### 2.B Impact of distance and depth on the bolus effect

To further evaluate the impact of distance and depth on dose reductions caused by the Superflab bolus, we measured doses 7, 10, and 15 cm away from the beam axis, at various depths and on the incident side, using the setup in Fig. 1 with and without Superflab boluses of various thicknesses placed on the phantom surface and out of the beam. A PTW Farmer‐type ion chamber (IC) (N30013 PTW Farmer^®^ Ionization Chamber, PTW, Freiburg, Germany) was used, and both 6 and 10 MV open (1000 MUs) and IMRT beams (900 MUs) (the same beams used in Section 2.A except the number of MUs) were tested.

### 2.C Bolus effect on breast cancer radiotherapy

**Figure 2:**
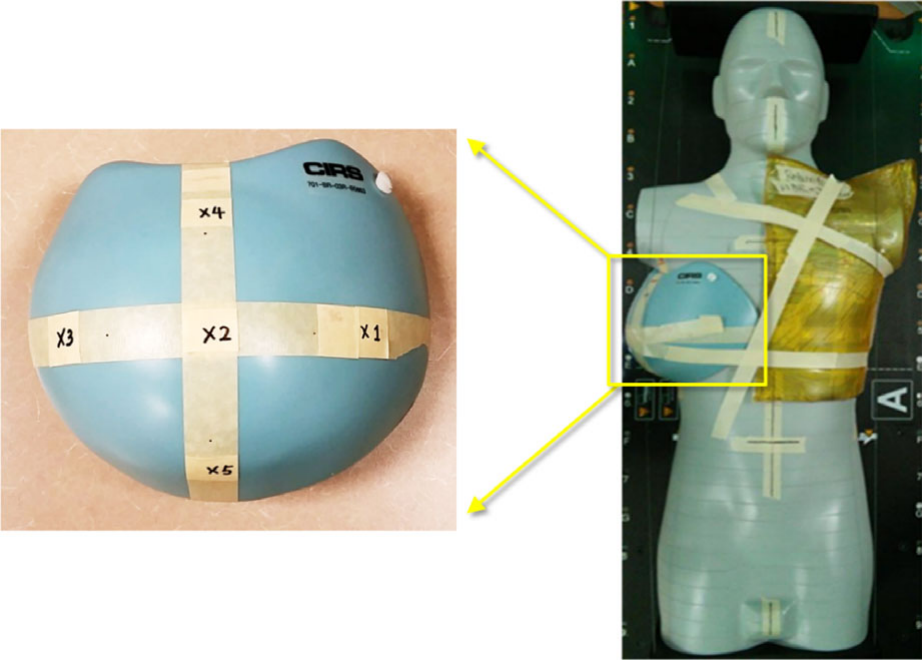
An Atom phantom (CIRS) with a breast attachment. Surface doses were measured on the contralateral breast attachment (points 1–5 in the left figure). A Superflab bolus was placed on the ipsilateral side to improve skin coverage. Another 1‐cm Superflab bolus was placed on the contralateral breast during radiation delivery to reduce surface dose but was not shown here for visual clarity.

To test the bolus effect in clinically realistic situations, we used an Atom dosimetry phantom (CIRS, Inc., Norfolk, VA, USA) for treatment planning and dose measurements (Fig. 2). The phantom was scanned by a GE LightSpeed 16 Slice computed tomography (CT) scanner (GE Healthcare, Little Chalfont, United Kingdom), and CT images with 2.5 mm slice thickness were imported into the Pinnacle 9.8 treatment planning system (TPS) (Philips Healthcare, Amsterdam, Netherlands) for treatment planning. Planning target volume (PTV) and organs at risk (OARs) were contoured in the TPS and were approved by a physician. Postmastectomy radiotherapy (PMRT) plans were generated including volumetric‐modulated arc therapy (VMAT), 4‐field IMRT, and Tomotherapy, with the prescription dose of 50 Gy in 25 fractions. For VMAT plan, dual‐arc with 230° rotations (between 180° and 310°) was used to cover the whole PTV. For 4‐field IMRT plan, three 6 MV IMRT beams with gantry angles of 295°, 315°, and 150°, and one 10 MV IMRT beam with gantry angle of 170° were used to cover the whole PTV. For Tomotherapy, CT images and contoured structures were exported from Pinnacle into TomoTherapy^®^ Hi∙Art TPS (Accuray, Madison, WI, USA), and a pitch of 0.287, a modulation factor of 2.8, 5.02 cm field width and fine dose grid were used for Tomotherapy optimization. For all treatment techniques, a 1‐cm Superflab bolus was placed on the ipsilateral breast to improve skin coverage. TLDs were placed at the measurement points to evaluate the surface dose reduction on the contralateral side, as shown in Fig. 2. The measurements were performed with and without placing a 1‐cm Superflab bolus on the contralateral breast attachment.

**Figure 3:**
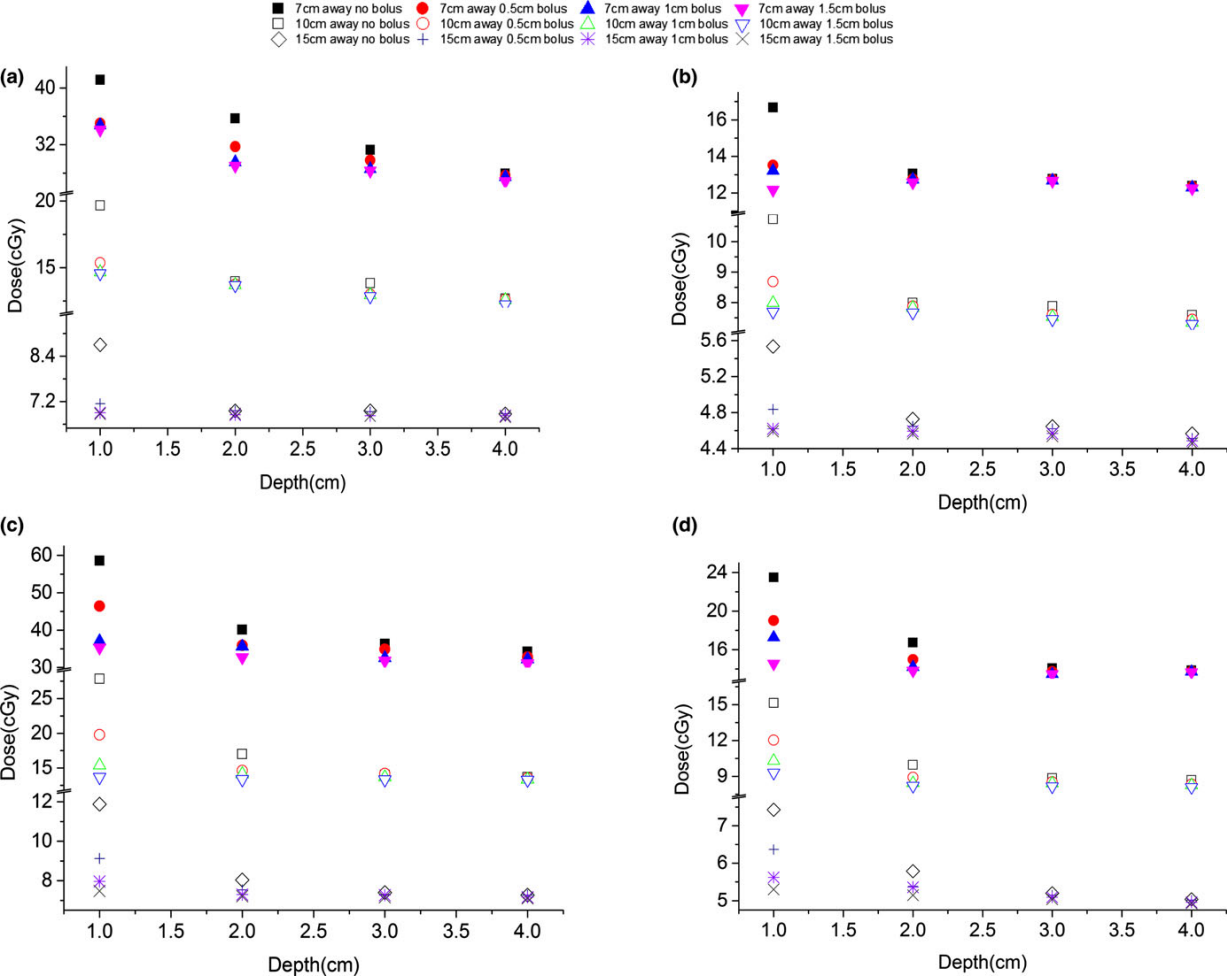
Out‐of‐field dose values at various off‐axis distances and depths with and without Superflab bolus of various thicknesses placed on top of the solid water phantom for: (a) 6 MV open field; (b) 10 MV open field; (c) 6 MV IMRT field; (d) 10 MV IMRT field. The measurement setup is shown in Fig. 1.

## 3 RESULTS AND DISCUSSION

The effects of Superflab bolus on surface dose are shown in Table 1. It is found that surface dose was reduced only when the point of interest is out of the beam and on the incident side of the beam (point 3 in Fig. 1 and Table 1), and the dose reduction increased with bolus thickness for both open and IMRT beams and for both beam energies. This is because the bolus placed on the surface absorbed the low energy head scatter and leakage photons and the thicker bolus absorbed more.

**Table 1:** Measured surface doses (mean ± SD of the mean) when Superflab boluses of various thicknesses were placed on a solid water phantom, and 6, 10 MV open (2000 MUs), and IMRT (1982 MUs) photon beams were delivered to the phantom. The setup and measurement point locations are shown in Fig. 1

Radiation beam	Point	Dose (cGy)				
		No	0.5 cm	1 cm	1.5 cm	2 cm
		Bolus	Bolus	Bolus	Bolus	Bolus
6 MV open	1	51.5 ± 0.8		139.3 ± 2.9		
	2	56.0 ± 3.2		63.6 ± 3.2		
	3	68.4 ± 1.5	49.8 ± 1.1	33.5 ± 0.5	24.7 ± 1.3	23.8 ± 0.4
	4	39.0 ± 0.3	41.0 ± 1.1	42.2 ± 0.7	44.5 ± 0.8	44.6 ± 0.7
6 MV IMRT	1	30.0 ± 0.8		68.4 ± 2.4		
	2	31.0 ± 3.4		40.0 ± 4.0		
	3	28.9 ± 1.0	23.4 ± 0.4	20.1 ± 0.7	17.7 ± 1.2	16.8 ± 1.1
	4	17.6 ± 0.4	18.4 ± 0.5	18.5 ± 0.8	19.6 ± 0.4	20.3 ± 0.9
10 MV open	1	51.0 ± 3.5		131.5 ± 2.8		
	2	63.1 ± 2.5		65.3 ± 3.4		
	3	77.8 ± 1.4	60.4 ± 1.3	40.5 ± 1.4	28.0 ± 0.9	24.2 ± 0.1
	4	36.7 ± 1.0	36.9 ± 1.6	38.4 ± 0.4	39.5 ± 1.8	39.5 ± 0.2
10 MV IMRT	1	35.2 ± 2.4		75.8 ± 2.0		
	2	35.0 ± 0.9		59.3 ± 3.4		
	3	35.5 ± 0.9	32.7 ± 1.9	31.9 ± 0.9	24.6 ± 0.7	20.9 ± 0.2
	4	10.4 ± 0.2	11.8 ± 0.2	11.9 ± 0.2	15.0 ± 1.9	16.5 ± 0.1

Doses increased at the other points: dose increment at point 1 (in‐field and on the incident side) was because the bolus moved the surface dose closer to the maximum dose point; dose increments at point 2 (in‐field and on the exit side) and point 4 (out‐of‐field and on the exit side) were due to the backscattering photons from the bolus and the backscatter increased with bolus thickness. However, the dose increments on the exit side (points 2 and 4) were relatively small compared with the dose reduction on the incident side (point 3). Therefore, for a treatment plan with multiple beam angles, the overall surface dose variation caused by the Superflab bolus would be determined by the beam angles from which the dose contributions are dominant.

Surface dose reductions in Table 1 are more significant for the open beam because the dose delivered to the calculation point was much higher from 2000 MUs open beam than from 1982 MUs IMRT beam. We scaled the number of MUs of 6 MV IMRT beam from 1982 MUs to 12413 MUs to match the dose from the open beam at the calculation point, i.e., to make them deliver the same amount of dose to the target like in the clinical radiotherapy, the doses at point 3 were 195.4 ± 5.9 cGy without bolus, 123.6 ± 2.9 cGy with 1‐cm bolus, and 103.2 ± 4.8 cGy with 2‐cm bolus on top (Table 2). As expected, the absolute dose reductions at the surface are more significant for the scaled IMRT beam because IMRT increases head scatter and leakage radiations. However, the relative dose reductions are still more significant for the open beam, which can be explained by the fact the linac jaws blocked more high energy photons than MLCs, while the Superflab bolus may not be able to completely absorb those high energy photons. Because this kind of delivery is very time consuming, we did not scale the 10 MV IMRT beam and did not measure at more locations with more bolus thicknesses.

**Table 2:** Measured surface doses (mean ± SD of the mean) when Superflab boluses of various thicknesses were placed on a solid water phantom, and 6 MV open (2000 MUs) and IMRT (12413 MUs) photon beams were delivered to the phantom. The setup and measurement point locations are shown in Fig. 1. The “no bolus” was used as the reference for dose difference calculations

Radiation beam	Point	No bolus	1‐cm bolus		2‐cm bolus	
		Dose (cGy)	Dose (cGy)	Difference (%)	Dose (cGy)	Difference (%)
6 MV open	3	68.4 ± 1.5	33.5 ± 0.5	−51.0	23.8 ± 0.4	−65.2
6 MV IMRT	3	195.4 ± 5.9	123.6 ± 2.9	−36.7	103.2 ± 4.8	−47.2

The effects of Superflab bolus on shallow depth dose are shown in Fig. 3. In out‐of‐field region and on the incident side, for both open and IMRT fields, the dose reductions in shallow depths caused by the bolus were more significant at closer distances from the radiation beam (Fig. 3). Most of the dose reductions occurred in the first 2‐cm depth and the dose reductions stopped at 4‐cm depth in all cases. These show that a Superflab bolus could effectively absorb scatter and leakage radiations and could reduce not only the surface dose but also shallow depth doses for both open and IMRT fields.

When the Superflab bolus was used for clinical PMRT beams, surface doses were reduced in most cases except Tomotherapy, as shown in Table 3. For VMAT plan, the surface dose reduced at most points and the reductions were between 13% and 31%, while the dose slightly increased 2% at point 3. The surface dose at point 3 decreased first during the beam delivery because the bolus was placed on the incident side of the beam and out of the field when the gantry faced the anterior part of the phantom, but the surface dose also increased because point 3 was located on the exit side of the beam when the gantry rotated to face the lateral and posterior part of the phantom. For the other points, the amount of dose reduction was greater than dose increment. For 4‐field IMRT plan, the dose reductions were greater than those in VMAT plan and the reductions were between 53% and 62%. However, the dose at point 1 increased because it was in the path of 295° beam and therefore the dose increased significantly. For the other points, the bolus reduced skin dose significantly because the contralateral breast was not included in the beam path or on any beam's exit side. Because Tomotherapy is characterized by full‐arc beams, any point on the surface will always be on both incident and exit sides of the beams and the benefit of using the bolus is diminished.

**Table 3:** Measured surface dose (mean ± SD of the mean) when 1‐cm Superflab bolus was placed on the contralateral breast of an anthropomorphic phantom and PMRT plans (VMAT, 4‐field IMRT and Tomotherapy) were delivered to the phantom. Doses, expressed in cGy, correspond to a total prescription dose of 50 Gy. The measurement locations are shown in Fig. 2

Point	VMAT			4‐field IMRT			Tomotherapy		
	No of bolus	With bolus	Difference (%)	No of bolus	With bolus	Difference (%)	No of bolus	With bolus	Difference (%)
1	323.1 ± 2.7	254.3 ± 2.2	−21	764.4 ± 7.1	1282.3 ± 22.3	68	517.1 ± 7.2	532.2 ± 18.8	3
2	127.3 ± 2.0	88.3 ± 1.7	−31	191.8 ± 3.0	78.5 ± 1.1	−59	208.0 ± 2.2	209.4 ± 3.0	1
3	62.4 ± 2.7	63.4 ± 0.7	2	70.9 ± 2.4	31.5 ± 0.3	−56	267.6 ± 8.3	271.9 ± 3.8	2
4	173.1 ± 4.3	140.6 ± 3.6	−19	216.3 ± 6.3	81.8 ± 1.7	−62	316.0 ± 3.4	320.5 ± 3.0	1
5	184.1 ± 2.5	160.1 ± 6.8	−13	77.0 ± 0.9	36.0 ± 1.4	−53	201.5 ± 2.2	212.7 ± 2.3	6

Reduction of undesirable doses from radiotherapy treatments represents an important topic due to the higher life expectancy after the treatments as a consequence of the high healing rate, increasing cancer incidence in the general population, and the increase in peripheral dose from new radiotherapy techniques.^34^ Literature demonstrates low radiation dose can induce severe side effects: it has been reported that atomic bomb survivors in the dose range from 5 to 100 mSv show a significantly increased incidence of solid cancer compared with the population who were exposed to less than 5 mSv,^35^ a significant risk for acute leukemia was seen in young individuals who were exposed to fallout from nuclear test site and received bone‐marrow doses from 6 to 30 mSv,^36^ thyroid and breast cancers occurred in children when radiation doses were as low as 100 mGy,^37^ and lung cancers happened for doses of 500 mGy in adults.^38^ The method presented in this study to reduce peripheral dose is a simple and important tool that could be used in clinical routine to reduce patients’ risk of developing radiation‐induced side effects and increase patients’ safety.

One limitation of this study is that we only evaluated this bolus effect for Elekta linac because this is the only type of linac in our clinic, while the other types like Varian machines may generate different results and investigation of this dose reduction strategy using other types of linacs will broaden the generality of our findings. However, the methodology of this study is generally applicable and clinics that have different types of machines could evaluate and validate this bolus effect easily using our approach. The other possible limitation is that we only validated this dose reduction strategy for advanced PMRT techniques, while conventional tangential technique or other cancer sites was not included. Tangential planning was already evaluated by Jamal and Das^31^ for whole breast irradiation and PMRT using Varian linac, and they reported the surface dose to contralateral breast could be reduced by 45% for the PMRT patient using 1‐cm Superflab bolus. For other cancer sites like head and neck, prostate, lung cancer radiotherapies, the bolus can also be used to shield the organs at risk as long as the bolus is out of the field and on the incident side of the beam.

## 4 CONCLUSIONS

We evaluated surface and shallow depth dose reductions under various conditions by placing the Superflab boluses of various thicknesses on a solid water phantom. The skin dose reduction caused by the bolus was achieved in out‐of‐field area and on the incident side of the beam, and the bolus effect increased with bolus thickness. The dose reduction caused by the bolus was more significant at closer distances from the beam. Most of the dose reduction occurred in the first 2‐cm depth and stopped at 4‐cm depth. We also evaluated this dose reduction strategy in clinically realistic situations by placing a 1‐cm Superflab bolus on the contralateral breast of an anthropomorphic phantom and delivered PMRT plans to it. The surface dose reduction was 13–31% for the VMAT plan and 53–62% for the 4‐field IMRT plan, but the bolus effect was not obvious for the Tomotherapy plan. In conclusion, using a bolus in out‐of‐field area and on the incident side of the beam can significantly reduce surface and shallow depth doses, but the absolute amount of dose change will be determined by the beam angles and how the radiotherapy plan was optimized. Although we only validated this dose reduction strategy for advanced PMRT treatments using one type of linac, it should be applicable to any external beam radiotherapy.

## CONFLICT OF INTEREST

The authors declare no conflict of interest.
